# A home-based, post-discharge early intervention program promotes motor development and physical growth in the early preterm infants: a prospective, randomized controlled trial

**DOI:** 10.1186/s12887-021-02627-x

**Published:** 2021-04-07

**Authors:** Juan Fan, Jianhui Wang, Xianhong Zhang, Ruiyun He, Shasha He, Mei Yang, Yujie Shen, Xiaojun Tao, Mei Zhou, Xiong Gao, Lijun Hu

**Affiliations:** 1 Department of Neonatology, National Clinical Research Center for Child Health and Disorders; Ministry of Education Key Laboratory of Child Development and Disorders; Chongqing Key Laboratory of Pediatrics, Children’s Hospital of Chongqing Medical University, Chongqing, China; 2 Department of Neonatal Surgery, Children’s Hospital of Chongqing Medical University, No 136, Zhongshan Er Road, Yuzhong District, Chongqing City, 400014 China

**Keywords:** Early preterm infants, Early intervention, Neurodevelopment, Physical growth

## Abstract

**Background:**

The implementation of early intervention (EI) in medical settings is time-consuming and resource-intensive, which limits its extensive use. In 2018, the Chinese Eugenics Association developed a home-based, post-discharge EI program. This study aims at evaluating the impact of this EI program on neurodevelopment and physical growth of early preterm infants.

**Methods:**

This study was a prospective, partially blinded, randomized controlled trial (RCT), followed by an open phase. A total of 73 infants born at 28^+ 0^ ~ 31^+ 6^ weeks’ gestation who were admitted to the Children’s Hospital of Chongqing Medical University between December 1, 2019, and June 31, 2020, were enrolled. Another 33 infants were retrospectively recruited as the reference group. Thirty-seven infants randomized in the first early intervention, then standard care (EI-SC) group performed a 30-day EI during RCT period, while 36 infants allocated to SC-EI group were given EI in the following open phase. The test of infant motor performance (TIMP), development quotient (DQ), and anthropometric measures (length, weight, head circumference) were measured at the baseline (T0), termination of the RCT (T1), and termination of the open phase (T2). Repeated measures analysis was performed for comparison among groups.

**Results:**

From T0 to T1, both groups had significant improvements in all outcome measures (all *p* < 0.001). A 30-day EI program was more effective in improving TIMP than standard care (from 53.12 ± 8.79 to 83.50 ± 11.85 in EI-SC group vs from 50.52 ± 8.64 to 75.97 ± 13.44 in SC-EI group, F = 4.232, *p* = 0.044). EI-SC group also had greater improvements in length, weight, and head circumference than SC-EI group (all *p* < 0.05). From T0 to T2, there was no significant difference regarding the improvements in all outcomes between the groups (all *p* > 0.05). At the endpoint of T2, the EI-SC and SC-EI group had similar TIMP and anthropometric measures, but much higher than the reference group (all *p* < 0.05).

**Conclusions:**

These findings demonstrated that a home-based, post-discharge EI program in this study was a practical approach to promote motor development and physical growth in early preterm infants.

**Trial registration:**

CHICTR, CTR1900028330, registered December 19, 2019, https:// http://www.chictr.org.cn/showproj.aspx?proj=45706

**Supplementary Information:**

The online version contains supplementary material available at 10.1186/s12887-021-02627-x.

## Background

In the last two decades, the survival of early preterm infants (EPI) has dramatically improved due to the tremendous progress of neonatal intensive care. However, these survivors’ high incidence of neurodevelopmental impairment is still of great concern to neonatologist and pediatricians [1]. Studies has reported that 53% ~ 72% of EPI experienced learning difficulties and had weaker performance in academics than their term peers, and 15% of them developed cerebral palsy [2, 3]. Currently, to improve the long-term outcome of EPI, particularly the neurodevelopment, is a significant challenge in the pediatric field.

Early intervention (EI) is believed to improve the neurological prognosis of infants born prematurely, who was at high risk for neurodevelopmental impairment [3–6]. Preterm infants have considerable brain plasticity during the early stage of life, and their nervous system has excellent development potential [7]. Applying EI to preterm infants can provide benign stimulus to the nervous system, modify the immature brain tissue, shape the brain structures, and ultimately improve the infant neurodevelopment [4–6, 8]. A large body of literature has also demonstrated the effect of EI on promoting the physical growth of infants, including weight, length, and head circumference [9, 10].

Most EI programs are currently carried out in the hospital, follow-up clinics, or community-based medical settings. Implementation of an EI program is laborious, time-consuming, and resource-intensive, limiting its use on a large scale, particularly in some medical resource-limited regions. In 2018, the Chinese Eugenics Association developed a home-based, post-discharge EI program, which aims to promote the global development of preterm infants. It was a revised version of the existing developmental intervention program for infants, which started in 2010 and was universal for either term or preterm infants. The current EI program makes parents the front-line executors of EI under the orientation of medical staff, and it seems to satisfy all requirements for an ideal EI model: flexibility, low-cost, and active involvement of infants and their families [11]. This program provides an innovative approach to carry out EI. To our knowledge, so far, no literature regarding the effectiveness of this EI program has been published internationally. This study was aimed to evaluate the impact of this home-based, post-discharge EI program on neurodevelopment and physical growth in EPI.

## Methods

### Study design

This study was a single-center, prospective, single blinded, randomized controlled trial (RCT) followed by an open phase. It was approved by the Institutional Review Board of Children’s Hospital of Chongqing Medical University (No.2019–216) and was registered in ChiCTR (ChiCTR1900028330), with the full protocol being available online. Written consent was obtained from the parents. Our study adhered to the CONSORT guidelines.

Randomization was performed using a computer-generated permuted block randomization sequence (block-size 4, 1:1 allocation). All enrolled infants were randomly allocated to either the first early intervention, then standard care (EI-SC) group, or first standard care, then early intervention (SC-EI) group. The twins were allocated to the same group in favor of parental care. During the RCT period, infants in the EI-SC group had the opportunity to have a 30-day EI exposure superimposed with the standard care, while those in the SC-EI group were only given standard care at this stage. Assessments were performed for all infants at baseline (T0) and 60 days later (T1, primary endpoint). In the following open phase, the infants initially allocated to the EI-SC group continued standard care, while those initially allocated to the SC-EI group received a 30-day EI due to ethical consideration. Repeated assessments were scheduled at 120 days of study (T2, second endpoint). All assessments were blinded to the assessors.

### Participants

All EPI admitted to the Children’s Hospital of Chongqing Medical University between December 1, 2019, and June 31, 2020, were eligible for this study. The inclusion criteria were as follows: (i) born at 28^+ 0^ ~ 31^+ 6^ weeks’ gestation, with postmenstrual age of 36^+ 0^ ~ 39^+6^weeks; (ii) stable vital signs, no oxygen requirement, good daily weight gain; (iii) either of the parents being able to take round-the-clock care for the infants at home.

The exclusion criterion was as follows: (i) small for gestational age; (ii) presumed brain injury, including intraventricular hemorrhage≥grade II, various degree of periventricular leukomalacia, neonatal seizure; (iii) congenital or acquired sensory deficits; (iv) presence of a major dysmorphic feature, or laboratory-confirmed chromosomal abnormality; (v) single-family, or parents with language barriers, neurodevelopment impairment, or other disabilities expected to interfere with the implementation of EI; (vi) infants were given various interventions from specialist simultaneously, such as an occupational therapist, physiotherapist, and speech therapist; (vii) parents declined to participate.

A sample of preterm infants who met the above criteria and had complete medical records were respectively recruited to provide a reference group. All eligible infants registered in our hospital follow-up dataset three months prior to our study were all recruited. The clinical data of these infants were extracted from the dataset.

### Implementation of the EI program

Once the parents signed the consent to participate in this study, a training session would be arranged soon. A didactic lecture was delivered to the parents to provide them with a holistic view of this EI program, followed by a simulation workshop. This EI program mainly consists of three sections: intellectual, physical, and social, which could be implemented separately. The intellectual section includes a hearing-induced training and a vision-induced training; the physical section involved a whole-body massage; the social section includes the kangaroo care and a hearing-vision integrated training (information about how to implement EI program was available in additional file 1). A researcher with rehabilitation backgrounds conducted all workshops. The parents were only eligible for performing EI independently after obtaining permission from the researcher.

Once the enrolled infants were discharged home, the parents should start the daily performance of EI promptly. Researchers would further assess the correctness of performance and suitability of the surrounding environment based on the videos obtained from the parents. Parents should also fill out an information card daily, recording the time and duration of EI, the operator, and infant response. A researcher was designated to collect such information.

### Standard care and follow-up clinic

Standard post-discharge care was provided to all eligible infants, including feeding guidance, strategies for illness and injuries prevention, bonding with the parents, scheduled immunization and available support service if necessary. The researchers recorded each sporadic clinic visit due to infant’s unwellness.

A bimonthly clinical follow-up was recommended for all early preterm infants. Pertinent advice and booklets dedicated to home-care were delivered to all parents. Each follow-up clinic visit took basic anthropometric measures, including weight, length, and head circumference (HC). All EPI received comprehensive neurological assessments due to their high risk for neurodevelopmental impairment. The follow-up information was stored in a hospital follow-up dataset.

### Outcome measures

#### Primary outcome measures

Test of Infant Motor Performance (TIMP) is developed to detect typical and atypical performance in preterm infants and evaluate the effect of an intervention on infant motor performance. A Chinese version of the TIMP test form, derived from the TIMP manual [12] and licensed by the Infant Motor Performance Scales, LLC (https://www.thetimp.com), was used in this study [13]. Assessments were performed by trained personnel certified by the Infant Motor Performance Scales, LLC.. TIMP consists of 42 items organized into two subsets (observed and elicited items). Observed items are designed to assess the infant’s spontaneous behavior (i.e., head orientation in the midline, ballistic movements of the limb), scored with one if found and 0 if absent. Elicited items are designed to assess the infant’s antigravity control and postural, auditory, and visual response to stimuli, scored with 0 to 6 based on the infant’s performance. A total raw score is summed from the individual item score, which was analyzed in this study. A higher score indicates better motor performance.

#### Secondary outcome measures

Gesell developmental schedules (GDS) were applied to all enrolled infants to check their developmental profile in five subdomains, including gross motor, fine motor, personal-social behavior, adaptive behavior, and language behavior [14, 15]. The Chinese version of GDS was translated and revised by the Beijing Mental Developmental Cooperative Group in 1985 and has been wildly used in China ever since. Developmental quotient (DQ) were calculated from GDS assessment, based on the infant’s performance on each subdomain (developmental age) compared to their chronological age at the time of assessment (DQ = developmental age/chronological age× 100). Each subdomain DQ, as well as the overall general DQ (the average of all subdomain DQs), were analyzed in this study. A score of more than 85 is regarded as average development.

The anthropometric measures, including weight, length, and HC, were also recorded and analyzed as the secondary outcomes in this study.

### Statistics

The sample size was calculated on the basis of a published reference [16]. A sample of 28 infants in each group was required to detect a clinically relevant change of 5 in TIMP raw score with a power of 80% at a significance level of 0.05 (allocation ratio = 1). Assuming a follow-up rate of 90%, the sample size was 31 in each group.

Clinical data were collected using Epidata 3.1 software, and all data analyses were carried out using SPSS 19.0. The normality of the data was evaluated using the Shapiro-Wilk test and the normal Q-Q plot. Normal distributed continuous data were expressed as the mean ± SD, which was compared using one-way ANOVA between groups at baseline (T0). The categorical data were expressed as a percentage, which was compared using the chi-square or Fisher’s exact test between groups. A *p* value< 0.05 was considered significant.

Repeated measures analysis was performed for the comparison of outcome measures from T0 to T1 between EI-SC group and SC-EI group to assess the effect of EI program versus standard care on infant development and physical growth. Furthermore, a similar analysis for outcomes from T0 to T2 was performed to test whether the 60-day delayed EI program had similar effects compared with the EI implemented initially.

A third group, retrospectively recruited, was taken into the analyses as a reference group. At the endpoint of T2, all outcome measures were compared among the EI-SC, SC-EI, and reference groups using the one-way ANOVA and the Bonferroni test as a post hoc multiple comparisons.

## Results

**Fig. 1:**
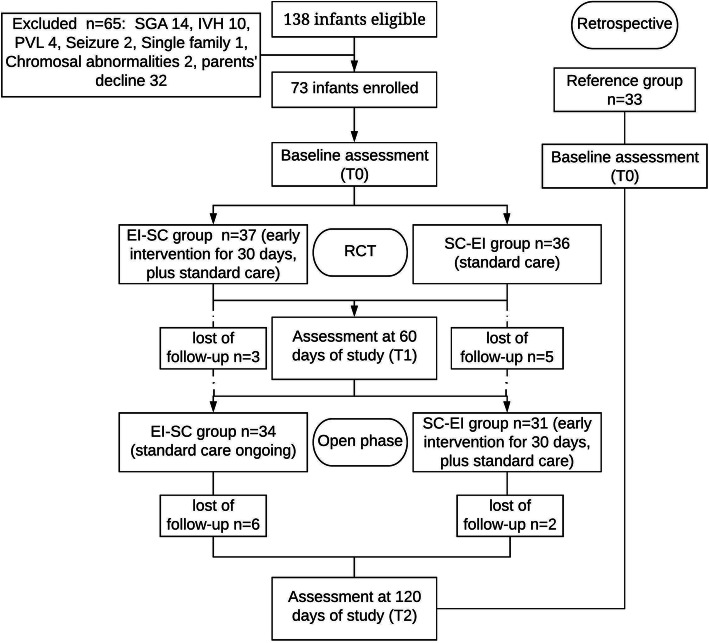
Study flow diagram. SGA, small for gestational age; IVH, intraventricular hemorrhage; PVL, periventricular leukomalacia; RCT, randomized controlled trials

A total of 138 infants were eligible for the RCT trial; 65 infants were excluded, resulting in 73 infants being randomly allocated, 37 in the EI-SC group (including one twin), and 36 in the SC-EI group. Besides, 33 infants were retrospectively recruited as the reference group (Fig. 1). No difference was noted among the three groups regarding demographic characteristics. The baseline TIMP, DQ, and anthropometric measures between the EI-SC and SC-EI groups were also comparable (Table 1).

**Table 1:** Baseline characteristics and assessment (T0)

	EI-SC group (*n* = 34)	SC-EI group (*n* = 31)	Reference group (*n* = 33)	*F* or *χ*2	p
Gestational age (weeks)	30.13 ± 1.56	30.35 ± 1.92	30.06 ± 1.39	0.273	0.762
Birth weight (gram)	1412.45 ± 178.34	1499.67 ± 200.23	1511.18 ± 187.06	2.744	0.069
Corrected gestational age (weeks)	36.87 ± 1.21	36.58 ± 0.92	36.97 ± 1.19	1.040	0.357
Weight at discharge (gram)	2499.73 ± 345.37	2492.11 ± 383.41	2504.94 ± 340.54	0.010	0.990
Female/male	24/10	18/13	19/14	1.544	0.462
Duration of maternal education (years)	14.45 ± 2.03	14.72 ± 2.56	14.82 ± 3.20	0.177	0.838
TIMP score	53.12 ± 8.79	50.52 ± 8.64	N/A	1.443	0.234
DQ overall general score	82.94 ± 5.85	79.32 ± 10.31	N/A	3.095	0.083
Gross motor	82.76 ± 14.90	78.84 ± 15.42	N/A	1.088	0.301
Fine motor	85.62 ± 20.47	78.71 ± 22.40	N/A	1.688	0.199
Adaptive behavior	83.21 ± 20.35	81.26 ± 31.20	N/A	0.090	0.765
Personal-Social behavior	80.29 ± 20.74	79.42 ± 17.40	N/A	0.034	0.855
Language behavior	83.24 ± 14.11	78.23 ± 14.25	N/A	2.024	0.160
Physical growth					
Length (cm)	47.26 ± 2.15	46.67 ± 1.90	47.04 ± 1.95	0.711	0.494
Weight (kg)	2.86 ± 0.84	2.73 ± 0.75	2.80 ± 0.65	0.243	0.785
Head circumference (cm)	32.06 ± 1.33	31.37 ± 1.65	32.21 ± 1.70	2.727	0.071

TIMP, the test of infant motor performance; DQ, development quotient, calculated from the Gesell Developmental Schedules assessment; N/A, no neurological assessment for the reference group at baseline (T0).

### From T0 to T1

Both groups had significant improvements in all outcome measures from T0 to T1 by repeated measure analysis (all *p* < 0.001). Furthermore, EI-SC group had significantly greater improvements in TIMP and physical growth (weight, length, and HC) than the standard care (all *p* < 0.05). However, no difference in the overall general DQ and each subdomain DQ was noted between groups (*p* > 0.05) (Table 2).

**Table 2:** Neurodevelopment score and physical growth from T0 to T1

	EI-SC group (n = 34)		SC-EI group (n = 31)		*F*	p
	T0	TI	T0	T1		
TIMP score	53.12 ± 8.79	83.50 ± 11.85	50.52 ± 8.64	75.97 ± 13.44	4.232	0.044
DQ overall general score	82.94 ± 5.85	87.12 ± 6.17	79.32 ± 10.31	83.00 ± 10.08	3.568	0.063
Gross motor	82.76 ± 14.90	88.06 ± 14.98	78.84 ± 15.42	83.26 ± 15.56	1.345	0.251
Fine motor	85.62 ± 20.47	87.50 ± 20.50	78.71 ± 22.40	80.84 ± 22.77	1.615	0.208
Adaptive behavior	83.21 ± 20.35	86.82 ± 21.07	81.26 ± 31.20	83.97 ± 31.10	0.136	0.713
Personal-Social behavior	80.29 ± 20.74	84.59 ± 20.94	79.42 ± 17.40	84.87 ± 16.88	0.004	0.950
Language behavior	83.24 ± 14.11	88.53 ± 15.46	78.23 ± 14.25	82.39 ± 14.55	2.388	0.127
Physical growth						
Length (cm)	47.26 ± 2.15	54.33 ± 2.85	46.67 ± 1.90	52.68 ± 2.41	6.229	0.015
Weight (kg)	2.86 ± 0.84	4.36 ± 0.95	2.73 ± 0.75	3.95 ± 0.79	4.095	0.047
Head circumference (cm)	32.06 ± 1.33	35.98 ± 1.86	31.37 ± 1.65	34.73 ± 2.20	6.133	0.016

TIMP, the test of infant motor performance; DQ, development quotient, calculated from the Gesell Developmental Schedules assessment;

### From T0 to T2

Both groups had significant improvements in all outcome measures from T0 to T2. However, no significant difference regarding the improvements in all outcomes between the EI-SC group and SC-EI group was noted (all *p* > 0.05, see additional file 2).

At the endpoint of T2, the EI-SC and SC-EI group had similar TIMP and anthropometric measures, which were significantly higher than that of the reference group by the Bonferroni test. In terms of the overall general DQ and each subdomain DQ, no difference was noted among the three groups (*p* > 0.05) (Table 3).

**Table 3:** Comparison of the outcomes at T2 assessment

	EI-SC group (*n* = 28)	SC-EI group (*n* = 29)	Reference group (*n* = 33)	*F* or *χ*2	p
TIMP score	113.54 ± 12.05^a^	109.33 ± 11.01^a^	101.18 ± 12.98^b^	8.332	<0.001
DQ overall general score	90.56 ± 6.14	87.08 ± 10.34	85.06 ± 7.14	1.538	0.130
Gross motor	94.50 ± 16.50	89.00 ± 17.71	84.94 ± 14.16	2.675	0.075
Fine motor	90.36 ± 21.88	83.10 ± 21.54	81.55 ± 15.63	1.673	0.194
Adaptive behavior	89.04 ± 19.13	90.55 ± 30.54	88.45 ± 21.00	0.062	0.940
Personal-Social behavior	88.57 ± 22.56	88.31 ± 18.73	85.70 ± 25.86	0.153	0.858
Language	90.57 ± 16.62	84.66 ± 13.74	84.67 ± 10.50	1.772	0.176
Physical growth					
Length (cm)	59.83 ± 2.77^a^	58.61 ± 2.61^a^	56.53 ± 2.45^b^	12.614	<0.001
Weight (kg)	5.56 ± 0.92^a^	5.24 ± 0.95^a^	4.74 ± 0.99^b^	5.547	0.005
Head circumference (cm)	38.91 ± 2.15^a^	39.38 ± 1.93^a^	38.20 ± 2.15^b^	5.492	0.006

^a, b^ denotes the significant difference by the Bonferroni test; TIMP, the test of infant motor performance; DQ, development quotient, calculated from the Gesell Developmental Schedules assessment;

## Discussions

EI means the intervention should be carried out as soon as possible, commonly started within the first 12 months of life. Post-discharge EI has been widely performed in follow-up clinics or in a community-based setting, which imposes high costs for the health care system. Taking Massachusetts, United States, as an example, the mean cost per infant for EI was 6614 dollars among the infants born at 24 ~ 31 weeks’ gestation, with a total EI cost of 65,910,379 dollars for all infants between 2009 and 2012 [17]. Such a huge number implied that the EI program might not be affordable in all countries, even in some developed countries. Meanwhile, some factors, such as traffic barriers, household overwork, and expensive accommodation cost, limit the parents to access the EI programs. Therefore, exploring a low-cost, family-based, flexible EI program was urgent for the medical providers and some families in need.

Amid this background, Sgandurra et al. developed a technological smart modular system as a tele-rehabilitation tool for EI, which could be implemented by parents at home under the orientation of rehabilitation staff. They demonstrated that this home-based EI significantly improved infant’s motor and visual development [16]. However, drawbacks also were evident; occurrence of technique errors seemed unavoidable for an internet-based system, and getting acquainted with a new system was also a challenge for some parents [18]. In this study, we conducted an innovative approach to carry out the home-based EI program, which was independent of any technological equipment and was free of knowledge gap for parents to perform it. Our study demonstrated that this EI program could promote motor development of EPI, as well as physical growth.

TIMP is the primary outcome measured in this study. Our results of the RCT period showed that the EI program was more effective in improving TIMP score than the standard care, which was consistent with Sgandurra’s study [16]; however, it conflicted with many previous studies, which did not show any significant effect of EI on motor development [4, 6, 19]. Some factors might contribute to the discrepancy. The frequency of EI performed in this study is once daily over 30 days, much higher than other studies which mostly ranged from once weekly to twice weekly [4]. Sgandurra et al. demonstrated that the amount of EI performance could affect the improvement of motor development [16] Meanwhile, our program adopted some promising evidence-based interventions, including body massage and kangaroo care. Body massage has been shown to effectively enhance motor development in various children population, including infants [20, 21]. The effect of kangaroo care on infant development was also well confirmed [22]. Massage and kangaroo care superimposed on basic intervention might generate a synergistic effect. Besides, different assessment tools might also lead to different results. Most previous studies used the Bayley physical development index (BPDI) to assess motor development, whereas our study used the TIMP instead. TIMP was approved to be a reliable and valid measurement of motor development, showing a highly significant correlation with BPDI [23]; TIMP is suitable for assessing infants < 6 months, while six months is the lower age limit for BPDI. Some studies have shown that the effect of EI on motor development was significant in infancy, but has not been noted for a long time. Therefore, a promising result in this study might be partially due to a relatively earlier assessment.

RCT did not show any significant difference in the DQ score between the EI-SC group and SC-EI group. Improvement of motor development demonstrated by TIMP was not present during the GDS assessments, which may be attributed to the structural limitation of the GDS. Each subdomain of GDS only has limited items under each month, scored as “observed” or “unobserved”; some minor changes cannot be specified. Meanwhile, GDS might delay identifying some subtle improvements, even some of which have already been noted by the parents [24]. In terms of physical growth, this EI program generally promoted the growth of weight, length, and HC, which was considered to be the result of a combination of many factors. Body massage has been shown to have the potential to improve gastrointestinal function and ultimately increase physical growth [25]. Literature also indicated that kangaroo care could shape preterm infant sleep behavior and prolong the duration of deep sleep [26]; a longer duration of deep sleep is a good predictor of optimal physical growth [27]. An EI program could also improve the infant’s feeding behavior, increase feeding volume, and positively affect physical growth [28, 29].

We conducted the open phase after the RCT, creditable from the consideration of ethics. No difference was noted between the EI-SC and SC-EI group regarding the improvements in all outcomes, which gave rise to the inference that the 60-day delayed EI had a similar effect to the EI performed at the beginning. Assessment at T2 endpoint also confirmed that no difference in TIMP and physical growth existed between the EI-SC and SC-EI group, but both were much higher than the reference group. It was an encouraging result for some parents who are concerned about the effect of the delayed intervention for their infants due to a variety of causes. However, currently, to carry out an EI program as soon as possible is still in recommendation.

Our study had certain limitations. We did not perform a long-term follow-up, hampering the evaluation of the long-term effect of this EI program. Meanwhile, the parent’s performance should alert the effectiveness of the EI program; however, it was impossible to keep all parents’ performances identical in this study. Fortunately, during the teaching session, our researcher should ensure that every parent had the basic skills to perform EI. In this study, we also cannot completely rule out the situation that some parents performed additional EI beyond the requirements of the study protocol, even the parents have initially signed the consent to comply with the study protocol.

## Conclusions

This study introduced a home-based, post-discharge EI program, which was less-cost, particularly suitable in medical source-limited regions. This EI program could significantly promote motor development and physical growth of EPI, at least in a short-term period. However, long-term follow-up is still in need to determine the long-term effect of this program on infant development.

## Supplementary Information

**Additional file 1.** information about how to implement EI program in the early preterm infants.

**Additional file 2.** Neurodevelopment score and physical growth from T0 to T2.

## Data Availability

The datasets used and/or analyzed during the current study, and the study protocol are available from the corresponding author on reasonable request.

## Abbreviations

EPI: Early preterm infants
EI: Early intervention
RCT: Randomized controlled trial
EI-SC group: First early intervention, then standard care group
SC-EI group: First standard care, then early intervention group
HC: Head circumference
TIMP: Test of infant motor performance
GDS: Gesell development schedules
DQ: Development quotient
BPDI: Bayley physical development index
