# Supplementary material for: A home-based, post-discharge early intervention program promotes motor development and physical growth in the early preterm infants: a prospective, randomized controlled trial
Source: BMC Pediatr. 2021 Apr 7;21:162. doi: 10.1186/s12887-021-02627-x (PMC8025314; doi:10.1186/s12887-021-02627-x)
Supplement: Supplementary file 1 — Additional file 1. information about how to implement EI program in the early preterm infants. [file 12887_2021_2627_MOESM1_ESM.docx]

**How to Implement Early Intervention in the Early Preterm Infants**

| **Sections** | **Training Items** | **How to Do?** |
| --- | --- | --- |
| INTELLECTUAL | Hearing-induced Training | When the baby is awakened quietly, hold baby’s head at a 45-degree angle with one hand, and gently shake a sand hammer 10 to 20 cm next to ear with the other hand; or, the mother gently calls the baby to turn around. From one ear to the other, take turns on both sides. Repeat 5 times, and take about 5 mins. |
|  | Vision-induced Training | When the baby is awakened quietly, hold the baby’s head at a 45-degree angle with one hand, and attract the baby’s sight with a red ball 20 cm away from the eyes using the other hand. Starting from the midline, move slowly to both sides, reaching 60-degree. Repeat 5 times, and take about 5 mins. |
| PHYSICAL | Whole-body Massage | Lay the baby on a table, apply massage oil on the trainer’s both hands, and perform the whole-body massage. (1)Head massage: 4 times from the front of the forehead to the back of the neck; (2) Facial massage: 4 times from the midline of the forehead to both sides; (3) Extremities massage: wrist upwards 4 times, and ankle upwards 4 times; (4) Chest massage: a circular massage from the bottom to the top, from the midline to both sides; (5) Abdomen massage: gently massage the abdominal wall 4 times. It takes about 10 mins. |
| SOCIAL | Kangaroo Care | Place the baby (naked except for a diaper and hat) on a mother’s or father’s chest (also bared to allow skin-to-skin touch) for up to a few hours. Lying position, sitting position, and standing position are all acceptable. It takes at least 2 hours. |
|  | Hearing-vision Integrated Training | Face the baby at a distance of about 20 cm and talk with the baby. If the baby is watching well, slowly move the baby’s head to both sides while talking to the baby. It takes about 10 mins. |

Relevant videos are available on YouTube with the hyperlinks as follows.

Hearing induced training:

<https://youtu.be/-fBgcv_Vy7o>

Vision-induced training:

<https://youtu.be/JIpOLQSJrSo>

Neonatal body massage:

<https://youtu.be/NtVDfuaG2Pk>

Hearing-vision integrated training:

<https://youtu.be/gobr6eDNfog>
