# Supplementary material for: A home-based, post-discharge early intervention program promotes motor development and physical growth in the early preterm infants: a prospective, randomized controlled trial
Source: BMC Pediatr. 2021 Apr 7;21:162. doi: 10.1186/s12887-021-02627-x (PMC8025314; doi:10.1186/s12887-021-02627-x)
Supplement: Supplementary file 2 — Additional file 2. Neurodevelopment score and physical growth from T0 to T2. [file 12887_2021_2627_MOESM2_ESM.docx]

|  | EI-SC group  (n=28) | | | SC-EI group  (n=29) | | | *F* | p |
| --- | --- | --- | --- | --- | --- | --- | --- | --- |
|  | T0 | TI | T2 | T0 | T1 | T2 |  |  |
| TIMP score | 52.57±8.80 | 82.89±11.18 | 113.54±12.05 | 50.83±8.82 | 76.07±13.83 | 109.33±11.01 | 2.986 | 0.090 |
| DQ overall general score | 82.25±6.07 | 86.57±6.51 | 90.56±6.14 | 79.24±10.37 | 82.97±10.12 | 87.08±10.34 | 2.243 | 0.140 |
| Gross motor | 83.11±15.27 | 88.36±15.12 | 94.50±16.50 | 79.48±15.56 | 83.86±15.67 | 89.00±17.71 | 1.176 | 0.283 |
| Fine motor | 85.21±21.61 | 87.07±21.68 | 90.36±21.88 | 77.28±21.78 | 79.34±22.12 | 83.10±21.54 | 1.761 | 0.190 |
| Adaptive behavior | 81.96±17.97 | 85.82±18.80 | 89.04±19.13 | 83.45±30.95 | 86.21±30.79 | 90.55±30.54 | 0.028 | 0.868 |
| Personal-Social behavior | 79.75±22.46 | 84.25±22.63 | 88.57±22.56 | 78.03±16.23 | 83.75±16.11 | 88.31±18.73 | 0.025 | 0.875 |
| Language behavior | 81.71±14.19 | 87.21±15.93 | 90.57±16.62 | 77.79±14.58 | 82.00±14.87 | 84.66±13.74 | 1.634 | 0.206 |
| Physical growth |  |  |  |  |  |  |  |  |
| Length (cm) | 47.16±2.35 | 54.21±3.03 | 59.83±2.77 | 46.61±1.95 | 52.59±2.47 | 58.61±2.61 | 3.707 | 0.059 |
| Weight (kg) | 2.76±0.86 | 4.24±0.94 | 5.56±0.92 | 2.70±0.76 | 3.93±0.81 | 5.24±0.95 | 1.131 | 0.292 |
| Head circumference (cm) | 31.93±1.39 | 35.82±1.92 | 38.91±2.15 | 31.83±1.70 | 35.25±2.27 | 39.38±1.93 | 0.751 | 0.390 |

Neurodevelopment score and physical growth from T0 to T2
